# JAB1/CSN5: a new player in cell cycle control and cancer

**DOI:** 10.1186/1747-1028-5-26

**Published:** 2010-10-18

**Authors:** Terry J Shackleford, Francois X Claret

**Affiliations:** 1 Department of Systems Biology, The University of Texas MD Anderson Cancer Center, 1515 Holcombe Boulevard, Houston, Texas 77030, USA

## Abstract

c-Jun activation domain-binding protein-1 (Jab1) acts as a modulator of intracellular signaling and affects cellular proliferation and apoptosis, through its existence as a monomer or as the fifth component of the constitutive photomorphogenic-9 signalosome (CSN5). Jab1**/**CSN5 is involved in transcription factor specificity, deneddylation of NEDD8, and nuclear-to-cytoplasmic shuttling of key molecules. Jab1/CSN5 activities positively and negatively affect a number of pathways, including integrin signaling, cell cycle control, and apoptosis. Also, more recent studies have demonstrated the intriguing roles of Jab1/CSN5 in regulating genomic instability and DNA repair. The effects of Jab1/CSN5's multiple protein interactions are generally oncogenic in nature, and overexpression of Jab1/CSN5 in cancer provides evidence that it is involved in the tumorigenic process. In this review, we highlight our current knowledge of Jab1/CSN5 function and the recent discoveries in dissecting the Jab1 signaling pathway. Further, we also discuss the regulation of Jab1/CSN5 in cancers and its potential as a therapeutic target.

## Introduction

Through detailed knowledge of oncogenic signal transduction pathways, targeted therapies have provided exciting advancements in the treatment of cancer where standard chemotherapy alone has failed. We discuss here a potential therapeutic target, the c-Jun activation domain-binding protein-1 (Jab1), which has been implicated to be involved in the tumorigenic process. Jab1 is involved in multiple protein interactions that affect many stages of tumorigenesis and, therefore, has the potential to be an effective therapeutic target.

Jab1/CSN5 was originally identified as a c-Jun coactivator and subsequently discovered to be the fifth member and an integral component of the constitutive photomorphogenic-9 (COP9) signalosome (CSN) complex, a multifunctional protein complex involved in modulating signal transduction, gene transcription, and protein stability in cells [1-3]. For this reason, Jab1 is also referred to as CSN5. Jab1/CSN5 contains an Mpr1-Pad1-N-terminal (MPN) domain metalloenzyme motif (JAMM) that is essential for the CSN isopeptidase activity responsible for the deneddylation of the cullin-RING ubiquitin ligases (CRLs) by CSN [4].

Jab1/CSN5 plays an essential role in positively regulating cellular proliferation by functionally inactivating several key negative regulatory proteins and tumor suppressors through their subcellular localization, degradation, and deneddylation, including p53, Smad 4/7, and the cyclin-dependent kinase inhibitor p27^Kip1 ^(p27) [5-8]. It is also capable of stabilizing certain proteins, including hypoxia-inducible factor 1a (HIF-1α) and c-Jun, as well as acting as a transcriptional co-factor for MYC, which is responsible for the transcriptional activation of genes involved in cell proliferation, angiogenesis, and invasion [1,9,10]. Jab1/CSN5 overexpression has been identified in a number of tumor types and has been implicated in the initiation and progression of several types of cancer.

### Jab1/CSN5 structure and function

**Figure 1:**
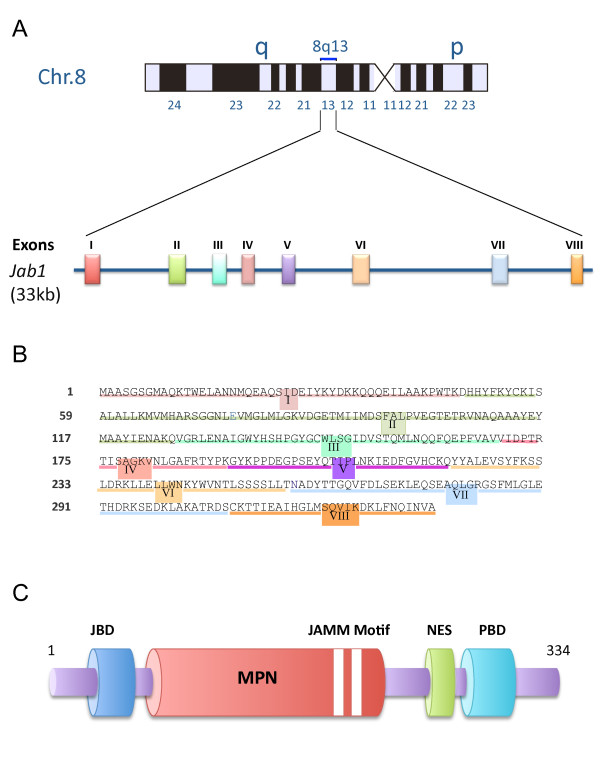
**Structure of the Jab1/CSN5 gene and domains of the Jab1/CSN5 protein**. (A) Schematic of the Jab1/CSN5 gene which is 33 kb long and localized at chromosome 8Q13.2. (B) The 8 exons within the 334 amino acid sequence are shown. (C) Schematic of Jab1/CSN5 consists of 334 amino acids with a Jab1/CSN5/MPN domain metalloenzyme (JAMM)-containing Mpr1-Pad1-N-terminal (MPN) domain and a nuclear export signal (NES) domain close to the p27 binding domain (PBD) at the C-terminal end.

The Jab1/CSN5 gene is highly conserved and extensively studied in mice. The murine *Jab1/CSN5 *gene is located on chromosome 2, whereas the human *Jab1/CSN5 *gene is located on chromosome 8 (Figure 1A). The human Jab1/CSN5 protein consists of 334 amino acids and has a molecular mass of 38 kDa (Figure 1A, B). While there are two *Arabidopsis *Jab1/CSN5 homologue genes, AJH1 and AJH2, there is only one known isoform in humans [11,12]. Jab1/CSN5 is evolutionarily conserved in humans, mice, fission yeast, and plants, which provides evidence that Jab1/CSN5 is critical to cell survival and proliferation [13-15]. Jab1/CSN5 was linked to the CSN through the discovery that the fifth member of the CSN was, in fact, Jab1/CSN5 and also when AJH1 and AJH2, which encode subunit 5 of the CSN, were identified in *Arabidopsis *[11,12,14]. Jab1/CSN5 has subsequently been termed the CSN subunit 5 (CSN5) [16].

Jab1/CSN5 is present in the large CSN holocomplex, smaller complexes, and as a monomer in *Arabidopsis*, *Drosophila*, and mammalian cells [8,12,17,18]. Whereas CSN-associated Jab1/CSN5 is located primarily in the nucleus, free-form Jab1/CSN5 can be both cytoplasmic and nuclear. Attempts to further study the presence of Jab1/CSN5 in these complexes have been made. Separation of the CSN and the smaller complex by gel filtration method or by the nondenaturing polyacrylamide gel electrophoresis (native-PAGE) technique has proven to be a valuable tool for analyzing the presence of Jab1/CSN5 in these complexes [8,17,19]. These studies have provided valuable information about the localization and stability of Jab1/CSN5; however, the overall picture of when Jab1/CSN5 as a member of the large or small complex or in its free form and the effect on various cellular processes remains to be studied.

The domains responsible for Jab1/CSN5's interaction with p27 and its metalloproteinase activity have been identified and are shown in Figure 1C. Jab1/CSN5 directly binds to p27 and mediates p27's shuttling between the nucleus and the cytoplasm in a CRM1-dependent manner through a nuclear export signal (NES)-like sequence between amino acids 233 and 242 at its C-terminal end [8]. Jab1/CSN5 has a Mpr1-Pad1-N (MPN) terminal domain that contains a Jab1/CSN5 MPN domain metalloenzyme (JAMM) motif (also known as the MPN+ motif). It has been postulated that the Jab1/CSN5 MPN domain serves as a protein-protein platform, while the JAMM motif acts as a cofactor for enzymatic activity [20]. The JAMM motif within Jab1/CSN5 appears to be key to CSN deneddylation activity, but deneddylation is still reliant on the entire CSN complex, as the loss of any one of the CSN subunits results in loss of this activity [20]. The JAMM motif is required for Jab1/CSN5's co-activation of the oncogenic MYC transcription factor and for Jab1/CSN5's transformative effects in a breast epithelial model, this was also found to be dependent of the assembly of the entire COP9 signalosome [21]. This domain does not, however, appear to be critical to Jab1/CSN5's functions outside of the full CSN, such as the stabilization of HIF-1α and its role as a co-factor for E2F1 apoptotic function [9,22]. In fact, the domains responsible for many of Jab1/CSN5's interactions and the mechanism by which it performs its many functions, as well as the involvement of the COP9 signalosome or small complex, remain to be determined. Elucidation of these domains will provide important insights that will further characterize the mechanisms of Jab1/CSN5 as well as the CSN.

### Jab1/CSN5 is a member of the CSN

The CSN is a multisubunit complex that controls protein stability by regulation of the CRL family and is also a regulator of many cellular and developmental processes, including cell-cycle control, transcription, and DNA-damage response (please refer to reviews for more detailed information [20,23-25]. The CSN consists of 8 subunits and forms a large, 450- to 550-kDa holocomplex as well as smaller (250- to 300-kDa) cytoplasm-localized complexes containing a subset of CSN subunits (CSN-4, -5, -6, -7b, and -8 in mammals) [8,17,26]. Jab1/CSN5 is one of two members of the CSN that contain the MPN domain.

Jab1/CSN5 is a key CSN subunit, able to integrate multiple functions of the CSN complex [3]. The CSN regulation of the CRL family of ubiquitin E3 complexes relies on its deneddylation function [23,27,28]. The JAMM domain present within Jab1/CSN5 which is the catalytic center for the CSN's cleavage of the ubiquitin-like protein Nedd8 from the Cul1 subunit of the SCF ubiquitin ligases [4] is the same domain present in the 26S proteasome lid component, RPN11, and is responsible for the proteasome's cleavage of the ubiquitin substrate [29,30]. Jab1/CSN5 alone does not have metalloproteinase activity; other CSN subunits, or perhaps the entire complex, are required for this deneddylase activity [4,23,25]. The JAMM domain also has deubiquitinase activity, which regulates ubiquitinated protein sorting into exosomes when Jab1/CSN5 is associated with the CSN [31]. This links the CSN with the regulation of ubiquitinated proteins that are transferred by exosomes, a process that impacts the flow of information from neighboring or distant cells.

### Jab1/CSN5 as a coactivator

Jab1/CSN5 was initially identified as a coactivator of c-Jun, a member of the activating protein-1 (AP-1) complex [1]. Members of the Jun (c-Jun, JunB, JunD) and Fos (c-Fos, FosB, Fra-1 and Fra-2) families form the AP-1 complex, which acts as a mitogen-activated composite transcription factor that has binding sites know as TPA (phorbol diester, 12-*O*-tetradecanoylphorbol-13-acetate)-responsive element (TRE) in a variety of promoters that activate various target genes. The AP-1 complex can respond immediately to many different extracellular stimuli, such as tumor-promoting TPA, epidermal growth factor (EGF), and serum [32]. Although c-Jun and c-Fos have very similar DNA binding and dimerization domains, they seem to activate distinct sets of target genes that only partially overlap [33-35]. Jab1/CSN5 can specifically stabilize the protein-DNA complexes on the off-rate of c-Jun and JunD with its cognate AP-1 DNA binding sites and potentiate c-Jun transactivation; however, Jab1/CSN5 does not affect JunB or v-Jun binding [1]. Jab1/CSN5's stabilization of c-Jun offers a mechanism of specificity in the binding of c-Jun to target genes.

Jab1/CSN5 is involved in c-Jun-induced, AP-1-mediated transcriptional activation through its interaction with c-Jun as well as affecting the interaction between c-Jun and other binding partners [1,36-38]. Jab1/CSN5 affects other proteins and their interaction with c-Jun by binding directly to and changing their sub-cellular localization. These interactions have been identified with the following proteins and in general lead to activation of AP-1: lymphocyte function-associated antigen-1 (LFA-1) [36,39], macrophage migration inhibitory factor (MIF) [40], hepatopoietin [41], and hepatitis B virus X protein (HBx) [42]. c-Jun is a well known oncogene whose aberrant expression and activity have been detected in cancer [32,43-46]. Because c-Jun expression is autoregulated, one can imagine that Jab1/CSN5, as a c-Jun-coregulator, may reinforce this positive-feedback loop at the transcriptional level and drive c-Jun protein expression further to promote tumorigenesis.

Jab1/CSN5's involvement as a specificity factor has been demonstrated for a number of proteins in addition to c-Jun. Jab1/CSN5 is a transcriptional coactivator and potential specificity factor for nuclear factor-kappaB (NF-κB), p53 binding protein 1 (53BP1), Brain-2 (Brn-2), and heart and neural crest derivatives expressed 2 (HAND2) signaling [37,47-50]. Jab1/CSN5 also has been shown to interact with the proto-oncogene Bcl-3, a member of the IκB family of inhibitory proteins and is present predominantly in the nucleus. While most members of the IκB family act to inhibit NF-κB, Bcl-3 can activate NF-κB transcription. Jab1/CSN5 binds to Bcl-3 to enhance NF-κB, p50, and DNA complex formation and may link NF-κB and AP-1 gene transcription [47]. Another example of Jab1/CSN5 as a specificity factor is its binding to the HAND2 HLH domain and stabilization of the HAND2-E-protein 12 complex on DNA and potential involvement in promoting gene expression during neuronal development [50]. Jab1/CSN5 was also found to bind directly to 53BP1 and, under mitotic stress conditions, is required for the hyperphosphorylation resulting in activation of the mitotic checkpoint mechanism [48]. Interaction of Jab1/CSN5 with Brn-2, a transcription factor expressed in the developing neocortex, was postulated to play a role in Brn-2-related neuronal development and, potentially, in the development of neurodegenerative diseases such as Parkinson's disease and Alzheimer's disease [49]. Jab1/CSN5 also acts as a bridge between coactivators and receptors in the progesterone receptor-steroid receptor coactivator complex [37].

These studies provide evidence of the role of Jab1/CSN5 as a specificity factor, yet the mechanism by which it acts is poorly defined. Phosphorylation is one mechanism of regulation that could be attributed to Jab1/CSN5's activation of kinases such as the Jun N-terminal kinase (JNK), which was reported to lead to increased phosphorylation of c-Jun, or through activation of kinases associated with the CSN [51,52]. Other transcription factors phosphorylated by the CSN include AP-1 and NF-kB. Also, the CSN itself has been characterized as a transcriptional regulator, recent data suggest that the CSN may be associated with chromatin [24]. Another mechanism involves the CSN's regulation of protein stability through ubiquitination of transcription factors. Whether Jab1/CSN5 functions independently as specificity factor or is mediated by functions of the COP9 signalosome has yet to be determined.

### Jab1/CSN5 a positive regulator of cell cycle progression

Jab1/CSN5 promotes cell proliferation by interacting directly with p27^Kip1 ^(p27) and induces nuclear export and subsequent p27-degradation. p27 is a critical component of the cell-cycle machinery [53]. As an inhibitor of cyclin E-Cdk2, p27 plays a pivotal role in controlling cell proliferation and therefore the cell's entry into S phase and exit from G1 phase during development and tumorigenesis [54,55]. Although p27 is haploinsufficient for tumor suppression, low levels of the protein have been identified in several cancers [56]. In addition, p27's cytoplasmic translocalization has been observed in human tumors and is associated with poor survival [57-61]. Jab1/CSN5's nuclear to cytoplasmic shuttling of p27 play an important role in the regulation and function of p27. Accumulation of cytoplasmic p27 results in increased cell motility and migration through its interaction with RhoA, a GTPase that regulates cell motility by reorganization of actin filaments [62,63]. Therefore Jab1/CSN5's role in p27 cellular localization interrupts p27's inhibitory effect on cellular proliferation and also puts it in position to interact with RhoA to mediate cell motility and migration which has an overall tumorigenic effect on the cell. In post-translational control, Jab1/CSN5--either alone or as a member of the CSN--plays an important role as a mediator of nuclear export and subsequent degradation of p27. Other Jab1/CSN5 interactions also act to enhance or inhibit Jab1/CSN5-mediated p27 degradation and are listed in Table 1.

**Table 1:** Jab1/CSN5-interacting proteins that regulate p27

Protein	Description	Interaction effect	References
PGP9.5	Ubiquitin C-terminal hydrolase	p27 degradation	[103]
Thioredoxin	Cellular redox enzyme that regulates cell growth and apoptosis	Inhibits Jab1/CSN5-mediated AP-1 transactivation and Jab1/CSN5-dependent p27 degradation	[104]
VDUP1	Tumor suppressor and stress response gene, vitamin D3 up-regulated protein-1	Inhibits Jab1/CSN5-mediated AP-1 transactivation and Jab1/CSN5-dependent p27 degradation	[105]
Hepatitis B virus pre-S2 mutant surface antigen	Hepatitis B virus large antigen mutant associated with hepatocellular carcinogenesis	p27 degradation	[106]

Abbreviations: PGP9.5, Protein gene product 9.5; AP-1, activator-protein 1; VDUP1, Vitamin D3 up-regulated protein 1

### Jab1/CSN5 as a modulator of intracellular signaling

**Figure 2:**
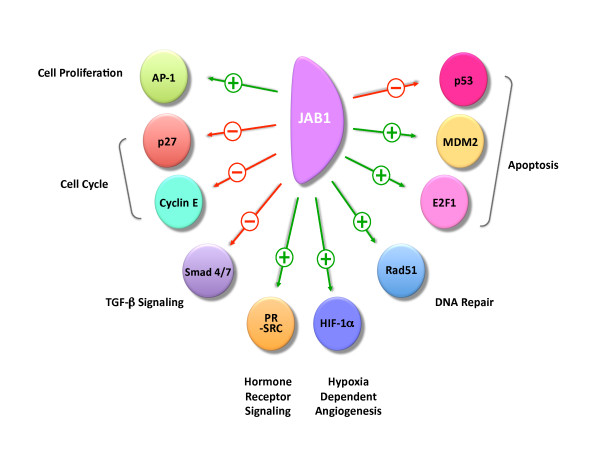
**Regulation and targets of Jab1/CSN5**. Green arrows and plus (+) signs indicate Jab1/CSN5-positive interaction; red arrows and minus (-) signs indicate Jab1/CSN5-negative interaction. Abbreviations: AP-1, activator protein 1; HIF-1α, hypoxia inducible factor-1 α; MDM2, murine double minute 2; PR, progesterone receptor, SRC-1, steroid receptor coactivator 1; TRC8, translocation in renal carcinoma

Jab1/CSN5 binds numerous proteins, generally resulting in cell proliferation, survival and in some cases angiogenesis and invasion. Table 2 summarizes these interactions that contributed to Jab1/CSN5's role in intracellular distribution, as a transcriptional activator, a member of the COP9 signalosome as well as novel Jab1/CSN5 interactions. Certainly, Jab1/CSN5's role in nuclear to cytoplasmic shuttling and subsequent degradation is widely known and affects many important proteins and tumor suppressors including p27, p53, and Smad 7, to name a few. Figure 2 outlines many of these interactions that act to positively or negatively regulate a number of signaling pathways in the cell.

**Table 2:** Proteins that interact with Jab1/CSN5

Protein	Description	Effect of Jab1/CSN5 interaction	Overall signaling effect	References
**Proteins degraded by Jab1/CSN5**				

p27	Cyclin-dependent kinase inhibitor and tumor suppressor	Nuclear export and degradation	Increased cellular proliferation	[8]
LHR	Lutropin/choriogonadotropin receptor	Degradation	Reproductive disorders	[38]
p53	Transcription factor and tumor suppressor	Nuclear export and degradation	Inhibit p53 tumor suppressor function	[6,107,108]
Smad 4	Co-Smad, positive regulator of TGFß signaling	Ubiquitination and degradation	Inhibit TGFß signaling	[7]
Smad 7	Inhibitory Smad, negative regulator of TGFß signaling	Nuclear export and degradation	Increase TGFß signaling	[69]
ERα	Estrogen receptor α	Degradation	Increase hormone induced transcription	[109]
West nile virus Capsid	Activates caspase-3 and caspase-9 in the apoptosis pathway	Nuclear translocation and degradation	Protective against West Nile Virus	[42]
Cyclin E	Cell cycle control, G1 to S phase	Degradation	Cell cycle	[110]
Rad9-Rad1-Hus complex	Involved in DNA damage sensing and DNA repair	Degradation	Impair DNA checkpoint and repair response to DNA damage	[70]
RUNX-3	Runt-related transcription factors	Nuclear export and degradations	Inhibition of a tumor suppressor	[111]
MIF	Cytokine with tautomerase and oxidoreductases activities	Inhibition of MIF secretion	Inhibits MIF-mediated AKT signaling	[112]
DNA topoisomerase (topo) II alpha	Enzyme that is essential for cell proliferation that segregates chromosome pairs during chromosome condensation	Degradation in a MPN dependent manner under glucose starvation	Decreased cell proliferation under stress conditions such as glucose starvation	[113]
Endothelin type A and B receptors	G protein-coupled receptors whose overexpression is correlated with chronic heart failure and in infiltrating cells of atherosclerotic lesions	Ubiquitination and degradation	Decreased Endothelin-1 induced intracellular signaling through ERK1/2	[114]

**Proteins affected by Jab1/CSN5**				

c-Jun	Member of the AP-1 transcription factor family	Transcriptional co-activator and specificity factor	Increased transcriptional activity and cellular proliferation	[1]
Myc	Oncogenic transcription factor	Promotes transcription of MYC target genes and induces MYC ubiquitination and turnover	Activates a wound signature and induced cell proliferation and invasion in breast cancer cells	[10]
HIF-1α	Hypoxia inducible factor α	Competes with p53 for binding, stabilizes protein HIF-1α levels	Increased expression of VEGF and angiogenesis	[9,115]
HAND2	Transcription factor important for development of the heart, limbs, and neural crest-derived lineages	Enhances HAND2 DNA binding	Tissue-specific transcription	[50]
53BP1	P53 binding protein, cellular response to stress conditions	Hyperphosphorylation under stress conditions	Activation of mitotic checkpoint mechanism	[48]
Smad 5	Receptor associated Smad protein, positive regulator of TGFß signaling	Inhibits bone morphogenetic signaling	Affect matrix turnover	[116]
Brn-2	POU transcription factor, development of neocortex and neural cell lineage	Increases Brn-2 transcriptional activity	Neuronal development and neurodegenerative diseases	[49]
Bcl-3	Member of Iκ-B family, proto-oncogene, can activate or inhibit NF-κB transcription	Bridges binding between Jab1/CSN5 and NF-κB	Link NF-κB and AP-1 gene activation	[47]
E2F-1	Transcription factor important for cell cycle progression, DNA damage repair, apoptosis	Cofactor for E2F-1 dependent apoptosis, but not cell cycle entry	Enhances E2F-1 mediated apoptosis	[22]
PR, SRC-1	Progesterone receptor, steroid receptor coactivator	Stabilized PR-SRC-1 complexes	Increased transcriptional activity	[37]
SMYD3	A histone methyltransferase	Suppressed transcription of the tumor suppressor p16	Negative regulation of p16 and possible increased in hematopoietic progenitors	[86]
Cullin	Subunit of SCF ubiquitin ligases	Cleavage of NEDD8 from Cul1	Required for optimal SCF ubiquitin ligase activity	[4]
PAR-2	G protein-coupled receptor for trypsin and tryptase	Increased PAR-2 transcription	Increased AP-1 activation	[117]
MDM2	Mediates p53 degradation	Reduces MDM2 self-ubiquitination	Negative regulation of p53	[108]
TRAF-2	TNFR-associated factor 2, mediator of TNFα prosurvival response	Ubiquitination of TRAF-2	Necessary for TNF-α prosurvival signaling and MMP production	[118]
Rad51	DNA repair protein involved in homologous recombination	Increases expression through negative regulation of p53	Increased ability of cell to repair DNA	[71]
FcαRI/CD89	Receptor for IgA expressed on myeloid cells and involved in phagocytosis, Ab-dependent cellular cytotoxicity, antigen presentation, and cytokine release	Binds directly to the intracellular domain and is involved in regulating stabilization of surface expression	Decreased expression of FcαR1 and possible defective antigen recognition response	[119]
5-HT(6)R	Serotonin receptor involved in the control of mood and emotion as well as involved in neurological disorders	Reduced Jab1/CSN5 expression decreases expression and activity	Reduced signaling through 5-HT(6)R, increased c-Jun activity and enhanced cell survival under hypoxia	[120]

Abbreviations: 5-HT(6)R, serotonin 6 receptor; AP-1, activator protein ; Bcl-3, B-cell lymphoma 3-encoded protein; ERα, estrogen receptor α; FcαRI, FCalphaRI; HAND2, heart- and neural crest derivatives-expressed protein 2; HIF-1α, hypoxia inducible factor α; LHR, lutropin/choriogonadotropin receptor; MIF, macrophage migration inhibitory factor; MDM2, murine double minute; NEDD8, neural precursor cell-expressed developmentally down-regulated; NF-kB, nuclear factor kappa-light-chain-enhancer of activated B cells; 53BP-1, p53 binding protein 1; PAR-2, abnormal embryonic PARtitioning of cytoplasm 2; PR, Progesterone receptor; SCF, Skp1-Cullin-F-box; SRC-1, steroid receptor coactivator 1; TRAF-2, TNF receptor-associated factor 2; VEGF, vascular endothelial growth factor

Jab1/CSN5 affects transforming growth factor-β (TGF-β) signaling by inducing degradation of two key downstream molecules, Smad4 and Smad7. Although TGF-β signaling can be tumor suppressive by inducing cell cycle arrest, differentiation, and apoptosis, it can be influenced by various factors in the tumor microenvironment as well as the tumor itself to, in fact, promote growth, invasion, and metastasis and contribute to the tumorigenic process [64,65]. The receptor-regulated Smad proteins (R-Smads) mediate TGF-β signaling through binding the common Smad, Smad 4, translocating to the nucleus, and mediating transcription of various genes [66]. Negative regulation of TGF-β signaling by inhibitor Smads (I-Smads), including Smad6 and Smad7, acts to interfere with the activation of the R-Smads [67,68]. Jab1/CSN5-induced Smad4 degradation results in reduced TGF-β-mediated gene transcription, whereas its degradation of Smad7 leads to enhanced TGF-β signaling effects [7,69]. It is therefore possible that connections exist between Jab1/CSN5 and TGF-β signaling that likely involve the COP9 signalosome. It is possible that overexpression of Jab1/CSN5 during the tumorigenic process results in enhanced TGF-β signaling that contributes to the progression of the disease.

Jab1/CSN5 has been implicated in apoptosis, DNA checkpoint and damage repair through a number of these protein interactions. Jab1/CSN5 can mediate the nuclear export and cytoplasmic degradation of the tumor suppressor p53 and enhances MDM2-mediated p53 ubiquitination [6]. It also mediates the degradation of the Rad9-Rad1-Hus (9-1-1) complex, thus impairing DNA checkpoint and repair in response to damage [70]. Recently, loss of Jab1/CSN5 results in spontaneous DNA breaks that was associated with increased expression of the histone H2AX, which recognized DNA double strand breaks and initiates recruitment of DNA damage repair proteins [71]. This is coupled with a deficiency in homologous recombination repair due to decreased Rad51 expression and function [71]. Taken together, these interactions provide evidence of Jab1/CSN5 as an inhibitor of DNA checkpoint and damage repair and an instigator of apoptosis. However, Jab1/CSN5's interaction with the transcription factor E2F-1 acted to synergistically induces apoptosis [22].

An interesting connection between Jab1/CSN5 and the hypoxia-inducible factor 1 alpha (HIF-1α) highlights Jab1/CSN5's potential involvement in the angiogenic pathway. HIF-1α is an oxygen-dependent transcriptional activator that is rapidly degraded under normoxic conditions, but under hypoxic conditions, it acts as a master regulator of a number of hypoxia-inducible genes, including those that are involved in angiogenesis and in cell proliferation, survival, and metabolism [72]. Jab1/CSN5 was found to compete with p53 to bind directly to the oxygen-dependent death domain of HIF-1α, leading to stabilization of HIF-1α by blocking hypoxia-dependent p53-mediated degradation [9]. In addition to stabilization of HIF-α, Jab1/CSN5 binding results in an increase in its transcriptional activity, resulting in increased VEGF expression [9]. This interaction between Jab1/CSN5 and HIF-1α suggest that Jab1/CSN5 is involved in the angiogenic pathway.

Taken together, many of these interactions are oncogenic and result in aberrant signaling involved in cell cycle progression, TGF-β signaling, angiogenesis, DNA checkpoint and repair, and apoptosis. These interactions themselves are often ill defined, and the role of the COP9 signalosome is not always clear. Further studies will need to be undertaken to identify the exact mechanisms taking place and to better define the role of Jab1/CSN5 in normal developmental processes as well as in tumorigenesis.

### Regulation of Jab1/CSN5's activity

One of the potentially oncogenic mechanisms of JAB1/CSN5 overexpression is through gene amplification by an increase in the DNA copy number. The JAB1/CSN5 locus is located on chromosome 8q13.1, a region that is frequently amplified in breast, prostate, colorectal and ovarian cancers [73-77], which has also been linked to aggressive cancer phenotypes and metastatic progression [10,78].

A few proteins have been shown to regulate Jab1/CSN5 levels, localization, or activity (Table 2). Psoriasin (S100A7), a small calcium-binding protein that is highly expressed in early breast cancer, enhances Jab1/CSN5 activity and promotes tumorigenesis [79,80]. Overexpression of psoriasin increases nuclear Jab1/CSN5 activity, resulting in increased AP-1 activity and reduced p27 expression [80]. The human epidermal growth factor receptor 2 (HER2) oncogene increases expression of Jab1/CSN5 through the AKT signaling pathway via the binding of the transcription factor β-catenin and transcription factor 4 (TCF-4) to the Jab1/CSN5 promoter [81]. Treatment with wortmannin, a phosphoinositide 3-kinase (PI3K)/AKT inhibitor, subsequently reduced Jab1/CSN5 expression [81]. Additionally, the oncogenic tyrosine kinase Bcr-Abl, which is a key contributor to the development of chronic myelogenous leukemia (CML), was found to be another key regulator of Jab1/CSN5 [82]. Bcr-Abl-expressing cells have higher levels of a cytoplasmic Jab1/CSN5-containing small complex were reduced on inhibition of the Bcr-abl kinase, the mitogen-activated protein (MAP) kinase, and the PI3K signaling pathways [82]. In another study, the peroxisome proliferator-activated receptor γ (PPAR γ) suppressed Jab1/CSN5 promoter activity in both PPAR γ-dependent and -independent manners [83]. These studies have linked Jab1/CSN5 expression and activity with well-known oncogenes and suggest that Jab1/CSN5 activities are involved in the progression of cancer. However, these oncogenes are fairly cancer specific and given the wide range of cancers that Jab1/CSN5 is found to be overexpressed, it is quite possible that other mechanisms or regulation of Jab1/CSN5 expression exists.

### Jab1/CSN5 in mouse models

Jab1/CSN5-deficient mice exhibit an embryonically lethal phenotype, which suggests that Jab1/CSN5 is important in development and survival. Jab1/CSN5-null embryos are viable up through the blastocyst stage but begin to exhibit disrupted development at embryonic day 6.0 and are no longer viable at day 8.5, before gastrulation occurs [71,84]. Several targets of Jab1/CSN5, including p27, p53, c-myc, and cyclin E, were found to be highly expressed in Jab1/CSN5^-/- ^embryos, resulting in impaired proliferation and accelerated apoptosis [71,84]. By building on these findings, we found that Jab1/CSN5 sensitized mouse embryonic fibroblasts to gamma radiation-induced apoptosis and increased spontaneous DNA damage that could be attributed to reduced levels of the DNA repair protein Rad51 and increased levels of p53 [71]. We found that in the absence of exogenous DNA damage, Jab1/CSN5-deficient embryos and osteosarcoma cells showed increased incidence of a spontaneous genome instability phenotype: findings included a large number of TUNEL foci in Jab1/CSN5-null embryos embryos and blastocysts and an increased number of γ-H2AX foci with a decreased percentage of intact DNA in Jab1/CSN5-deficient mouse embryonic fibroblasts and in human osteosarcoma cells [71]. These findings suggested that Jab1/CSN5-deficient cells promote spontaneous DNA breaks; therefore, loss of Jab1/CSN5 may affect efficient DNA repair. The results of these studies indicate that Jab1/CSN5 is essential for efficient DNA repair and mechanistically link Jab1/CSN5 to the maintenance of genome integrity and to cell survival.

Jab1/CSN5 was conditionally deleted to determine its role in cell cycle regulation, apoptosis, and DNA repair processes that are critical for developing thymocytes [85]. Conditional deletion of Jab1/CSN5 in thymocytes resulted in defective S-phase progression and increased apoptosis that could be attributed to CSN substrates, including p53, IκB-α, and β-catenin. These results confirmed the importance of Jab1/CSN5 in coordinating the process involved in the development and positive selection of T cells. Further, these findings provided some support for the conceptual premise that Jab1/CSN5, along with the rest of the CSN, orchestrates the development of various tissues by maintaining the balance between proliferation and survival throughout various checkpoints in the process.

Overexpression of Jab1/CSN5 in a transgenic mouse model was linked to myeloproliferative disease. Those mice showed enhanced proliferation and maintenance of hematopoietic progenitors [86]. Inversely, the results from additional studies suggest that inhibition of Jab1/CSN5 by siRNA results in delayed tumor growth in murine xenografts, a finding that justifies further investigation of Jab1/CSN5 as a potential therapeutic target [87].

### Jab1/CSN5 in cancer

Jab1/CSN5 has been implicated in the pathogenesis in several cancer types and in many cases, specifically correlated with reduced levels of p27 and poor prognosis. Because of emerging recognition of its role in negatively controlling p27 activity, recent pre-clinical studies have implicated Jab1/CSN5 function in the pathogenesis of human cancers. Tumors in which Jab1/CSN5 overexpression was detected are listed in Table 3. Low levels of p27 protein have been associated with high expression of Jab1/CSN5 in a number of tumors (Table 3). Low p27 levels have been reported in up to 50% of all human cancers, yet no modifications in the gene have been identified, and inhibition of p27 is postulated to occur at the post-transcriptional level [88,89]. In addition to low levels of p27 expression characterizing many tumors, studies have increasingly pointed to subcellular localization of p27 through cytoplasmic translocation in human tumors associated with poor survival rates [57-61,90,91].

**Table 3:** Cancers in which Jab1/CSN5 is overexpressed and its association with p27 and clinical outcome

Tumor type	Associated with poor outcome	Negative Correlation with p27	References
Pituitary tumor	No	Yes	[121]
Pancreatic adenocarcinoma	Yes	Yes	[100,122]
Oral squamous cell carcinoma	Yes	Yes	[123,124]
Epithelial ovarian tumors	Yes	Yes	[125,126]
Neuroblastoma	ND	ND	[127]
Embryonal rhabdomyosarcoma	No	Yes	[128]
Hepatocellular carcinoma	ND	Yes	[83,97,107]
Intrahepatic Cholangiocarcinomas	No	No	[129]
Laryngeal squamous cell carcinoma	Yes	Yes	[130]
Esophageal squamous cell carcinoma	Yes	Yes	[131]
Metastatic melanoma	ND	Yes	[132]
Invasive breast carcinoma	Yes	Yes	[92,93]
ERalpha-negative breast cancer	No	No	[98]
Systemic anaplastic large cell lymphoma	ND	Yes	[133]
Non-Hodgkin's lymphoma	Yes	Yes	[134]
Malignant lymphoma of the thyroid	ND	Yes	[135]
Thyroid medullary carcinoma	No	Yes	[136]
Papillary Thyroid Carcinoma	Yes	ND	[137]
Non-small-cell lung cancer	Yes	Yes	[138,139]

Abbreviation: ND, not determined

Jab1/CSN5-mediated degradation of p27 appears to be a critical mechanism of regulation for this cell cycle inhibitor. The correlation between high Jab1/CSN5 levels and low p27 levels in a number of tumors provides further evidence that this is a common phenomenon and may contribute to the pathogenesis of these diseases. For example, Jab1/CSN5 expression was found low or absent in normal adult breast tissue, but is aberrantly expressed in 50% of primary breast tumors and 90% of metastatic lesions making an ideal target for therapeutic intervention [92,93]. Our group examined Jab1/CSN5 and p27 protein expression in invasive breast carcinoma specimens and their association with clinical outcome [92,93]. We immunohistochemically detected Jab1/CSN5 in 43 (81%) of 53 tumors, with 32 (60%) tumors having high levels of Jab1/CSN5 expression (>50% of cells positive for Jab1/CSN5), but reduced or absent p27 expression (*P *= 0.02). Breast tumors with high levels of p27 expression were rarely positive for Jab1/CSN5 expression. Furthermore, Jab1/CSN5 protein expression levels were higher in oncogenically transformed breast cells and tumors than in normal mammary epithelial specimens. Furthermore, adenovirally mediated Jab1/CSN5 overexpression in breast cancer cells reduced p27 expression levels by accelerating p27 degradation. Importantly, patients with Jab1/CSN5-negative tumors had no evidence of relapse or disease progression at a median follow-up duration of 70 months [92]. These results correlate with the known molecular function of Jab1/CSN5 in relocalizing p27 from the nucleus to the cytoplasm, thereby promoting degradation of p27 through the ubiquitin/proteasome pathway, allowing breast tumor cells to progress into S phase.

There were also a small number of cases in which high Jab1/CSN5 expression did not correlate with low p27 expression. Other factors, such as the ubiquitin ligase, S-phase kinase-associated protein 2 (Skp2), are known to regulate p27 and have also been found to correlate with p27 expression in cancer. In one study, Skp2 expression correlated with reduced p27 in epithelial ovarian tumors, and although Skp2 alone was associated with poor prognosis, evaluation of the combined phenotype, Skp2(+) Jab1/CSN5(+) p27(-), led to identification of patients with the worst prognosis [94]. Given the importance of p27 degradation in cancer, development of a therapy to inhibit Jab1/CSN5 is likely to be clinically valuable.

While the connection between Jab1/CSN5 overexpression and reduced p27 is evident in human tumors, the correlation between Jab1/CSN5 and c-Jun activation, while apparent in preclinical studies, has yet to be validated in human specimens. Jun is a well-characterized oncogene that has been demonstrated to promote cellular proliferation and invasion in certain tissues [95,96]. Expression of c-Jun is evident and has proven to have prognostic value in a number of tumor types and to contribute to carcinogenesis [43-46]. Further, c-Jun is also downstream of a number of signaling cascades and therefore contributes to the process of oncogenesis even when expressed at normal levels [34]. While the link between Jab1/CSN5 overexpression in tumors and c-Jun activation has not been clearly identified in patient samples, our preclinical studies suggest that this correlation exists. High AP-1 activity was detected in Jab1/CSN5 overexpressing xenograft models (data not shown). Future studies to investigate this correlation in tumor samples would be useful in validating these findings.

Importantly, Jab1/CSN5/CSN5, alongside Myc, was found to act as a master regulator of a wound gene expression signature in breast cancer cells. This study suggests that Jab1/CSN5 plays an important role in translating the cell stress response to transcription of response genes that are involved in proliferation and matrix invasiveness [10]. Jab1/CSN5's location on chromosome 8q, which is frequently amplified during the progression of cancer and its function as a master regulator alongside Myc highlight the possibility that Jab1/CSN5 is a candidate oncogene. This DNA copy number gain of Jab1/CSN5 was confirmed in a study of hepatocellular carcinoma that correlated with amplification of the 8q region [97]. This suggests that the location of Jab1/CSN5 in this highly amplified region may be one mechanism leading to its overexpression in cancer.

Jab1/CSN5's role in breast cancer is increasingly becoming clearer. Studies have demonstrated Jab1/CSN5 overexpression in breast cancer [92,98,99]. The breast cancer driving EGFR receptors including the oncogenic HER2/neu have been correlated with increased Jab1/CSN5 expression in clinical samples [98,99]. Jab1/CSN5 was shown to be a target of EGFR signaling in estrogen receptor-alpha-negative (ERα^-^) breast cancer and the translocation of Jab1/CSN5 to the nucleus was shown to be mediated through the ERK signaling pathway [98]. Similarly, HER-2/neu was found to activate Jab1/CSN5 expression through the AKT/bet-catenin pathway [99]. The involvement of Jab1/CSN5 in breast tumorigenesis appears to be linked to the expression of EGFR and HER-2/neu receptors and may in part mediate downstream signaling events that contribute to the progression of tumor growth.

The detection of Jab1/CSN5 overexpression in a variety of human tumors suggests that it is a significant contributor to the tumorigenic process. Results from preclinical studies (and unpublished data) also provide evidence that Jab1/CSN5 is playing an important role in this process (references in Table 3). In a recent study by Adler et al, they investigated the ability of Jab1/CSN5 to promote breast epithelial transformation and also linked the biochemical activities of Jab1/CSN5. They found that Jab1/CSN5 indeed had transforming capabilities that was dependent on both the assembly of the COP9 signalosome and also the isopeptidase activity present in JAMM motif of Jab1/CSN5 [21]. Jab1/CSN5 is found to be overexpressed in a number of human tumors, but whether this overexpression is sufficient to mediate the tumorigenic process was tested in a transgenic mouse model. Intriguingly, mice overexpressing a modified Jab1/CSN5 show that the level of Jab1/CSN5 expression is a critical determinant of the proliferation and maintenance of hematopoietic progenitor cells in vivo, which may explain how the overexpression of Jab1/CSN5 contributes to tumor development [86]. It was postulated that perhaps an initial DNA damaging event, activation of an oncogene or alternatively inhibition of a tumor suppressor could be necessary for Jab1/CSN5 to mediate its tumorigenic effects. Additional studies suggest that depletion of Jab1/CSN5 is able to reduce proliferation in pancreatic cancer cells [100]. These data suggest that overexpression of Jab1/CSN5 contributes to the development of tumors and that inhibition of Jab1/CSN5 is sufficient to impact on proliferation and potentially other properties of tumors and warrants further investigation as a cancer therapeutic.

## Conclusion

Jab1/CSN5's multiple functions affect a number of proteins and signaling pathways, and the results of these interactions are generally oncogenic. The strong correlation between Jab1/CSN5 overexpression and low p27 levels and poor prognosis in a variety of human cancers underscores the importance of Jab1/CSN5 in carcinogenesis. However, the exact role that Jab1/CSN5 plays in oncogenic processes and which of its many functions contributes to these processes is far from being fully understood. It would be interesting to determine whether Jab1/CSN5 contributes to tumorigenesis through one specific interaction or the accumulation of multiple interactions. Further, whether these interactions are dependent on Jab1/CSN5's existence as either a member of the COP9 signalosome holo-complex or a member of smaller complexes outside the COP9 signalosome, which contain other members of the CSN, or its presence as a monomer, has yet to be clarified.

The physiologic significance of Jab1/CSN5 in cancer is highlighted by the wide range of cancers in which it is overexpressed. Yet the molecular mechanism by which Jab1/CSN5 is involved in the myriad of protein interactions remains to be more clearly identified. No particular protein domain identified within these proteins links them in any way. The JAMM domain is certainly important for cleavage of cullin-Nedd8 conjugates and represent a major player in cellular regulation, but it does not appear to be the key domain for all Jab1/CSN5 interactions. It has been suggested that a post-translational modification or possibly a redox-induced protein modification leads to a Jab1/CSN5 interaction; this warrants further investigation [101]. A link between the Ras/MAPK and PI3K/AKT pathways, which were detected through Bcr-Abl signaling, has been identified. Understanding the mechanisms of activation will be critical for understanding the regulation of Jab1/CSN5's functions. These mechanisms also involve interactions between the CSN and other large protein complexes as have been demonstrated with Iκ-B kinases and suggested to occur with nuclear receptors and their coactivators [37,102].

Much remains unclear about Jab1/CSN5, but what is clear is that its overexpression is involved in the development of cancer and that further investigation of Jab1/CSN5 as a therapeutic target may lead to the development of a powerful cancer therapeutic for use in a wide range of tumors.

## Conflict of interests

The authors declare that they have no competing interests.

## Authors' contributions

All authors have contributed to the writing of this paper. They have read and approved the final manuscript.
